# Flavonoid and Non-Flavonoid Compounds of Autumn Royal and Egnatia Grape Skin Extracts Affect Membrane PUFA’s Profile and Cell Morphology in Human Colon Cancer Cell Lines

**DOI:** 10.3390/molecules25153352

**Published:** 2020-07-23

**Authors:** Valeria Tutino, Isabella Gigante, Rosa Anna Milella, Valentina De Nunzio, Riccardo Flamini, Mirko De Rosso, Maria Principia Scavo, Nicoletta Depalo, Elisabetta Fanizza, Maria Gabriella Caruso, Maria Notarnicola

**Affiliations:** 1 Laboratory of Nutritional Biochemistry, National Institute of Gastroenterology “S. de Bellis” Research Hospital, 70013 Castellana Grotte (BA), Italy; valeria.tutino@irccsdebellis.it (V.T.); isabella.gigante87@gmail.com (I.G.); valentinadx@hotmail.it (V.D.N.); 2 Research Centre for Viticulture and Enology, Council for Agricultural Research and Economics, 70010 Turi (BA), Italy; rosaanna.milella@crea.gov.it; 3 Research Centre for Viticulture and Enology, Council for Agricultural Research and Economics, 31015 Conegliano (TV), Italy; riccardo.flamini@crea.gov.it (R.F.); mirko.derosso@crea.gov.it (M.D.R.); 4 Personalized Medicine Laboratory, National Institute of Gastroenterology “S. de Bellis” Research Hospital, 70013 Castellana Grotte (BA), Italy; maria.scavo@irccsdebellis.it; 5 Institute for Chemical-Physical Processes (IPCF)-CNR SS Bari, 70125 Bari (BA), Italy; n.depalo@ba.ipcf.cnr.it (N.D.); elisabetta.fanizza@uniba.it (E.F.); 6 Dipartimento di Chimica, Università degli Studi di Bari Aldo Moro, 70126 Bari (BA), Italy; 7 Ambulatory of Clinical Nutrition, National Institute of Gastroenterology “S. de Bellis” Research Hospital, 70013 Castellana Grotte (BA), Italy; gabriella.caruso@irccsdebellis.it

**Keywords:** flavonoids, non-flavonoids, membrane PUFAs profile, cell morphology, human colon cancer cells

## Abstract

Grapes contain many flavonoid and non-flavonoid compounds with anticancer effects. In this work we fully characterized the polyphenolic profile of two grape skin extracts (GSEs), Autumn Royal and Egnatia, and assessed their effects on Polyunsaturated Fatty Acid (PUFA) membrane levels of Caco2 and SW480 human colon cancer cell lines. Gene expression of 15-lipoxygenase-1 (15-LOX-1), and peroxisome proliferator-activated receptor gamma (PPAR-γ), as well as cell morphology, were evaluated. The polyphenolic composition was analyzed by Ultra-High-Performance Liquid Chromatography/Quadrupole-Time of Flight mass spectrometry (UHPLC/QTOF) analysis. PUFA levels were evaluated by gas chromatography, and gene expression levels of 15-LOX-1 and PPAR-γ were analyzed by real-time Polymerase Chain Reaction (PCR). Morphological cell changes caused by GSEs were identified by field emission scanning electron microscope (FE-SEM) and photomicrograph examination. We detected a different profile of flavonoid and non-flavonoid compounds in Autumn Royal and Egnatia GSEs. Cultured cells showed an increase of total PUFA levels mainly after treatment with Autumn Royal grape, and were richer in flavonoids when compared with the Egnatia variety. Both GSEs were able to affect 15-LOX-1 and PPAR-γ gene expression and cell morphology. Our results highlighted a new antitumor mechanism of GSEs that involves membrane PUFAs and their downstream pathways.

## 1. Introduction

Grape (*Vitis vinifera* L.) is a fruit rich in polyphenols, bioactive compounds able to prevent the occurrence of cancer, reduce tumorigenesis, and influence important cancer-related pathways [1,2,3]. The anticancer effects of polyphenols are closely related to their chemical structure and concentration, as well as to the type of cancer [1,2]. Polyphenolic compounds are mainly divided into two groups: flavonoids, based on the common C6-C3-C6 skeleton which consists of two phenyl rings (A and B) linked by a heterocyclic ring (C), and non-flavonoids such as stilbenes (C6-C2-C6) and phenolic acids (C6-C1) [3,4]. The most abundant classes of flavonoids present in the grape skin and seeds include anthocyanins, flavonols, flavan-3-ols, and proanthocyanidins [5]. Flavonoids exist either as glycosides with attached sugars or as aglycones with no attached sugars, and differ in the degree of hydroxylation and substitution. These functional hydroxyl groups mediate the antioxidant effects of flavonoids and their ability to interact with biological membranes [2,6]. Stilbenes, such as resveratrol, are phytoalexins synthesized by plants in response to mechanical injury, UV irradiation, and fungal attacks [5]. Benzoic acid and cinnamic acid represent the most common phenolic acids present in the grape skin [7]. 

The phenolic composition of the grapes mainly depends on genotype but can be affected by environmental factors and agronomic practices [5]. Several in vivo and in vitro studies have demonstrated the antimetastatic effects of some polyphenolic compounds [8,9]. Mantena S. K. et al. evaluated the chemoprotective efficacy of grape seed proanthocyanidins in both metastatic breast cancer cells and in Balb/c mice, a mouse model of breast cancer obtained after subcutaneous implantation of the highly invasive and metastatic 4T1 mouse breast cell line [10]. There are several mechanisms of action through which grape polyphenols are able to inhibit the invasion and progression of metastasis [11,12,13]. These natural compounds can act on the structural components of the cytoskeleton, on cellular adhesions, and on the composition of the membrane fatty acids [11,14].

Polyphenols are able to influence cell membrane fluidity and cell motility by the stearoyl-CoA desaturase-1 (SCD1) enzyme activity, given by the oleic acid/stearic acid ratio [15,16]. The increase in the oleic acid content in cell membranes, and consequently the up-regulation of SCD1 enzyme, are known to stimulate the process of invasion and metastasis in human cancer cells [17,18]. Rearrangements in the lipidomic profile of the membrane are an important feature that distinguish cancer cells, since phospholipids are directly involved in the morphological changes occurring in tumorigenesis and tumor progression [16,19,20]. Moreover, it is now known that an imbalance in the ratio of omega-6/omega-3 (n-6/n-3) Polyunsaturated Fatty Acids (PUFAs), in favor of n-6, is associated with the development of chronic inflammatory diseases, including colon cancer [21,22]. Tumorigenesis of the colon is strongly influenced by the oxidative metabolism of PUFAs regulated by different enzymes, as 15-lipoxygenase-1 (15-LOX-1) [23,24]. 15-LOX-1 enzyme is known to exert antioxidant and antimetastatic action by activating peroxisome proliferator-activated receptor gamma (PPAR-γ) [25,26]. The preferred substrate for 15-LOX-1 is the essential fatty acid, namely linoleic acid (LA). This n-6 fatty acid with its metabolite, the 13-HODE (13-*S*-hydroxyoctadecadienoic acid), are down-expressed in human colon cancer [27].

Based on these assumptions, the main aims of the present study are: (1) to fully characterize the polyphenolic content of two table grape skin extracts (GSEs), Autumn Royal, a seedless black grape with healthy properties, and Egnatia, a new red seedless genotype obtained by breeding programs carried out by our research group; (2) to evaluate the effects of two GSEs on the membrane PUFA profile in two human colon cancer cell lines at different grade of differentiation, Caco2 and SW480; (3) to evaluate in the same treated cells the gene expression of 15-LOX-1 enzyme and its downstream factor PPAR-γ, as well as the possible changes in cell morphology.

## 2. Results and Discussion

### 2.1. Total Polyphenolic Content in Autumn Royal and Egnatia GSEs

**Figure 1:**
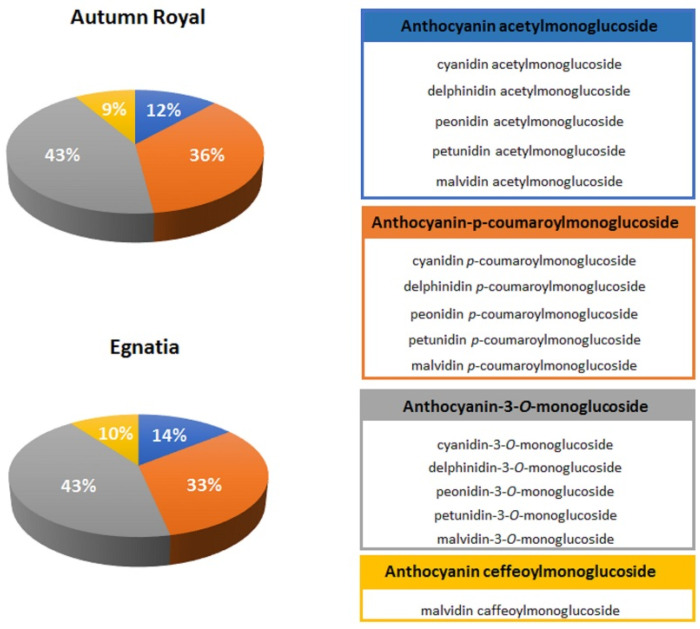
Anthocyanin composition of Autumn Royal and Egnatia table grape skin extracts expressed as the relative percentages (%) of M^+•^ signals intensity in the positive-ion Ultra-High-Performance Liquid Chromatography/Quadrupole-Time of Flight mass spectrometry (UHPLC/QTOF) chromatogram.

**Figure 2:**
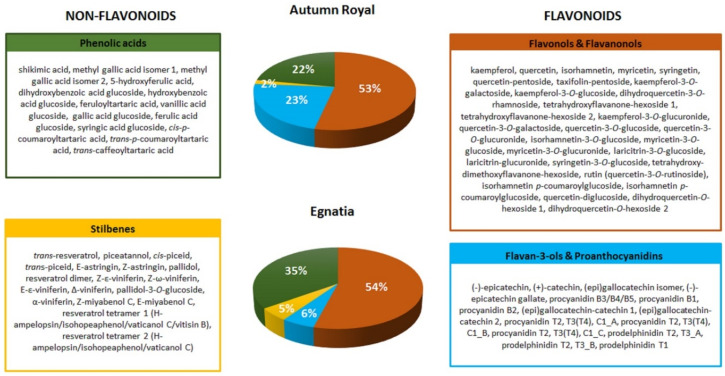
Flavonoid and non-flavonoid composition of Autumn Royal and Egnatia table grape skin extracts expressed as the relative percentages (%) of the total [M − H]^−^ signal intensity in the negative-ion Ultra-High-Performance Liquid Chromatography/Quadrupole-Time of Flight mass spectrometry (UHPLC/QTOF) chromatogram.

The total content of polyphenols was determined in Autumn Royal and Egnatia GSEs by a spectrophotometric assay. The results obtained confirmed that Autumn Royal is richer in total polyphenols, expressed as milligrams of gallic acid equivalents per gram of dry weight of skin (GAE/g dw), than Egnatia (53.10 ± 1.99 mg GAE/g dw versus 37.45 ± 0.73 mg GAE/g dw, *p*-value < 0.05, respectively) and that the anthocyanins were the most represented polyphenols in both varieties [28]. Polyphenolic profiles were determined by Ultra-High-Performance Liquid Chromatography/Quadrupole-Time of Flight mass spectrometry (UHPLC/QTOF) analysis. Anthocyanins were identified by positive ionization (Figure 1), the other polyphenols in negative ionization mode (Figure 2). Main MS/MS fragments and mean signal intensity of the metabolites identified in the samples are reported in the supplementary materials (Table S1). Positive and negative extract ion chromatograms (EIC), showing the signals of compounds identified, are reported in the supplementary materials (Figures S1A and S1B, respectively). More than 100 flavonoid and non-flavonoid compounds were identified in the both GSEs. 

The anthocyanin profiles of Autumn Royal and Egnatia GSEs were very similar and characterized by a higher percentage of anthocyanin-3-*O*-monoglucoside compounds (43% in both samples) (Figure 1). These molecules have different antitumor effects depending on the B-ring substituents [29]. Anthocyanins, whose structure contains an *o*-dihydroxy (catechol) B-ring, such as cyanidin-3-*O*-monoglucoside and delphinidin-3-*O*-monoglucoside, are characterized by tumorigenesis inhibition activity [30].

Signals of phenolic acids were relevant in both varieties (22% and 35% for Autumn Royal and Egnatia, respectively), while total stilbene signals intensity was 2% in Autumn Royal and 5% in Egnatia extracts (Figure 2). Both samples showed flavonols and flavanonols as main signals, with total signal intensity 53% and 54% in Autumn Royal and Egnatia, respectively. In particular, high intensity of the B-ring trisubstituted flavonols, such as myricetin and syringetin glycosides, was found. Instead, signals of flavan-3-ols and proanthocyanidins were higher in Autumn Royal (23%) compared to Egnatia extract (6%). Several studies showed that flavan-3-ol oligomers (proanthocyanidins) are potent antioxidants and free radical scavengers also characterized by anticancer properties [31,32]. In our previous study, we found a higher antioxidant activity in Autumn Royal compared to Egnatia, and this result could also be due to the greater content of flavan-3-ols and proanthocyanidins found in Autumn Royal GSEs [28].

Within each class of flavonoids, a structural variation exists in their basic 15-carbon skeleton, leading to different physicochemical properties. An improvement in the antitumor biological activities of flavonoids is due to the 3′- and 4′-hydroxyl groups (*o*-diphenol groups), known as catechol groups, present on the B-ring [33]. Compared to the Egnatia variety, Autumn Royal presented a higher catechol percentage, calculated by summing the intensities of the signals of those metabolites whose structure includes one or more *o*-diphenol groups (100% versus 57.6%, respectively). Table 1 shows the *o*-diphenol compounds identified in both GSEs.

The number and position of hydroxyl groups also influence the interactions of flavonoids with the cell membrane lipid bilayer. The hydrophilic flavonoids, which contain more hydroxyl groups, interact with the polar head groups by hydrogen bonds, inducing membrane rigidification. On the contrary, the hydrophobic flavonoids pass through cell membranes, causing a modification of their permeability and fluidity [6,34,35]. Therefore, the contribution of each phenolic compound can be different, and their synergistic/antagonist interactions might influence their biological effects.

### 2.2. Membrane PUFA Profile After GSE Treatment in Human Colon Cancer Cell Lines

To investigate the effects of Autumn Royal and Egnatia GSEs on the n-3 and n-6 PUFAs membrane composition, Caco2 and SW480 human colon cancer cell lines were treated with increasing concentrations of GSEs (20, 50 and 80 µg/mL), and the lipidomic profile was analyzed after 48 h of treatment (Table 2a,b). The choice to use these GSEs concentrations and this experimental time (48 h), was dictated by our previous study, in which we demonstrated that the greatest antiproliferative effect of GSEs in Caco2 and SW480 cell lines was observed in these experimental conditions [16].

Compared to the untreated control group (CTR), in Caco2 cells, the treatment with Autumn Royal and Egnatia GSEs caused an increase of both essential fatty acids (EFAs), linoleic acid (LA) and α-linolenic acid (ALA), already starting from the concentration of 20 μg/mL (Table 2a), and these increases did not correspond to a modification in the n-3 and n-6 fatty acids downstream pathways (Table 2a). Moreover, the increase in total PUFA levels, already at the lowest concentration (20 μg/mL) of both GSEs, was essentially due to the contribution of LA and ALA (Table 2a).

In the SW480 cell line, the treatment with Autumn Royal GSE increased LA levels at 20 µg/mL of concentration, whereas for Egnatia GSE, a higher concentration (50 µg/mL) was necessary (Table 2b). Compared to CTR, only a decrease in arachidonic acid (AA) levels was observed after exposure of both GSEs at 50 μg/mL (Table 2b). As regards the n-3 PUFAs pathway, ALA levels were induced after exposure to the two grape polyphenols, in particular after treatment with 80 μg/mL of Autumn Royal GSE and 20 μg/mL of Egnatia GSE (Table 2b). The increase in total PUFAs obtained after Autumn Royal treatment, at the concentration of 20 µg/mL, was due exclusively to the contribution of LA (Table 2b). The reduction of AA levels found at the concentration of 50 μg/mL of Autumn Royal was more pronounced than the increase of LA obtained at the same concentration of extract, thus balancing the levels of total PUFAs at 50 and 80 μg/mL (Table 2b).

Moreover, no alteration in n-6/n-3 ratio levels was found in all two cell lines and with both treatments (data not shown).

We used two human colon adenocarcinoma cell lines with different degrees of differentiation, Caco2 and SW480, in order to investigate the variations of the membrane PUFA levels induced by the quality of polyphenols contained in the two table grape varieties used. Cancer cells are in continuous proliferation and need large quantities of fatty acids and phospholipids to generate new cellular membranes [19,36]. Previously, we demonstrated the ability of Autumn Royal and Egnatia GSEs to influence membrane fluidity in Caco2 and SW480, through the inhibition of the enzyme SCD1 [16]. This enzyme, that converts saturated fatty acids (SFAs) into monounsaturated fatty acids (MUFAs), was reduced mainly in the Caco2 cell line after GSEs treatment. In this study, we demonstrated that Autumn Royal and Egnatia GSEs were also able to influence the membrane levels of total PUFAs. Different basal levels of PUFAs were found in untreated Caco2 and SW80 cells, probably due to cell type, developmental and growth stage of cells [37]. In SW480 cells with a lower degree of differentiation, the high AA levels found contribute to increasing the total PUFAs levels present in these cells, preparing the cellular pathways towards more inflammatory outcomes. 

Both GSEs induced the levels of EFAs, LA and ALA, in all two cell lines studied. EFAs are important structural components of cell membranes that the animal cells must exclusively obtain from their environment [37]. Therefore, the increase of LA and ALA detected in treated Caco2 and SW80 cells was certainly attributable to the fatty acids contained in the grape skins.

Several studies have shown that LA, γ-linolenic acid (GLA), and dihomo-γ-linoleic acid (DGLA) have anticancer effects, unlike AA, which has been associated with the inflammation and with onset of the tumor [38,39,40,41]. Treatment with increasing concentrations of both extracts increased LA levels in SW480 cells. In addition, in these cell lines, both extracts led to a reduction in AA compared to untreated cells, exerting an anti-inflammatory effect inside cells. As regards the n-3 fatty acids pathway, no variation was observed. Hanikoglu A. et al. found differences in the reorganization of fatty acids in cell membranes of two different breast cancer cell lines, MCF-7 and MDA-MB231, after treatment with somatostatin, curcumin, and quercetin, alone or in combination [14]. Cancer cells, according to the degree of differentiation, behave differently to treatment with drugs and/or natural compounds, and this feature could explain the different response of SW480 to treatment with GSEs, with respect to Caco2 cells.

### 2.3. Effects of GSE Treatments on the Gene Expression of 15-LOX-1 and PPAR-γ in Human Colon Cancer Cell Lines

**Figure 3:**
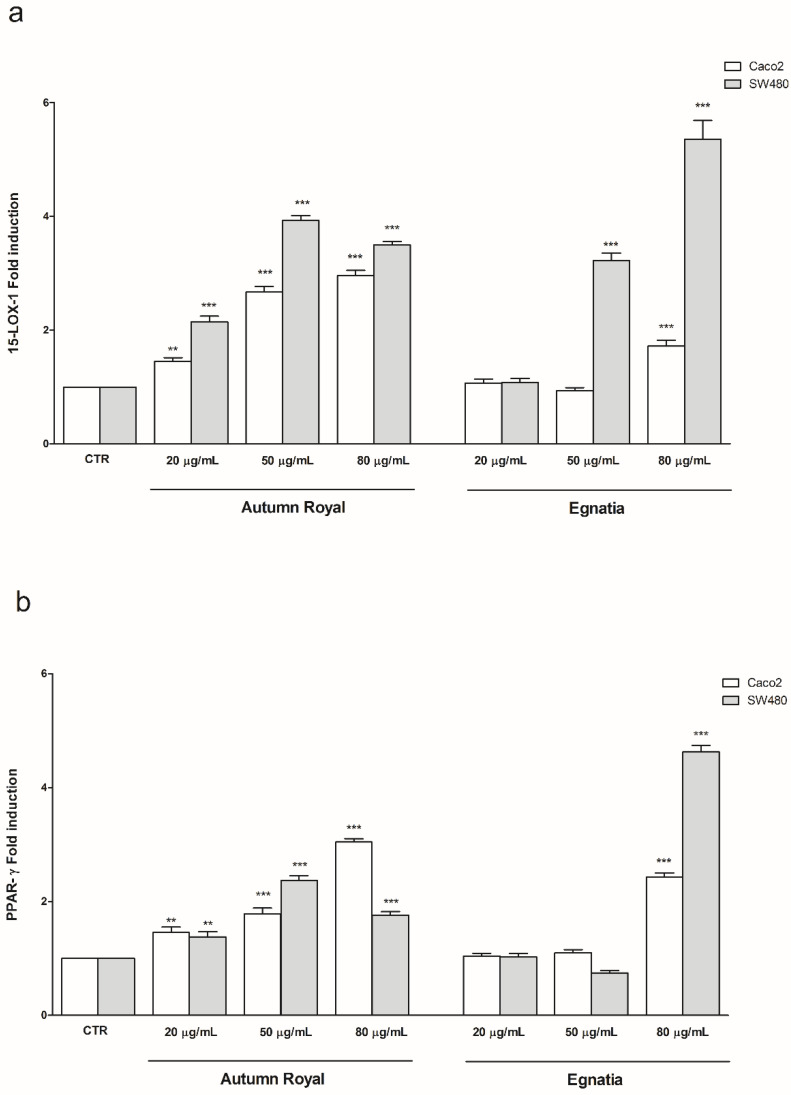
(**a**) 15-LOX-1 gene expression levels detected in Caco2 and SW480 cells treated with increasing concentrations (20, 50, 80 µg/mL) of Autumn Royal and Egnatia GSEs for 48 h of incubation; (**b**) PPAR-γ gene expression levels detected in Caco2 and SW480 cells treated with increasing concentrations (20, 50, 80 µg/mL) of Autumn Royal and Egnatia GSEs for 48 h of incubation. All data are expressed as mean ± Standard Deviation (SD) of three consecutive experiments. *p*-value was determined by ANOVA with Dunnett’s posttest. ** *p* < 0.03 and *** *p* < 0.01 versus untreated control group (CTR).

To better investigate the effects of Autumn Royal and Egnatia GSEs on membrane PUFA levels and their antitumoral and antimetastatic action, the gene expression of 15-LOX-1 and PPAR-γ, markers involved in the onset of colorectal cancer (CRC), was studied. Figure 3 shows the effects of increasing Autumn Royal and Egnatia GSE concentrations (20, 50, and 80 μg/mL) on the mRNA levels of 15-LOX-1 (Figure 3a) and PPAR-γ (Figure 3b) in Caco2 and SW480 cell lines after 48 h of treatment. Compared to CTR, Autumn Royal GSE induced a significant up-regulation of 15-LOX-1 gene expression in both cell lines studied, starting from the lowest concentration (20 μg/mL) (Figure 3a). Regarding treatment with Egnatia GSE, in Caco2 cells a higher concentration (80 μg/mL) was needed to observe a statistically significant increase in the gene expression levels of 15-LOX-1 compared to CTR (Figure 3a). For SW480 cells, the increase in the gene expression of 15-LOX-1 was already visible at 50 μg/mL of Egnatia GSE (Figure 3a). 15-LOX-1, through its product 13-S-HODE, activates PPAR-γ by inhibiting colorectal tumorigenesis. Therefore, possible changes in PPAR-γ gene expression after exposure to increasing concentrations of GSEs were investigated. Compared to CTR, Autumn Royal GSE treatment exerted an up-regulation of PPAR-γ mRNA levels, starting from 20 μg/mL in both Caco2 and SW480 cells (Figure 3b), whereas a higher concentration of Egnatia (80 μg/mL) was need to obtain the same significant increase in PPAR-γ gene expression (Figure 3b).

Carcinogenesis is known to be also caused by changes in PUFA levels of the cell membrane, including colon cancer formation [19]. 15-LOX-1 is able to oxygenate both n-3 and n-6 PUFAs. The main substrate of 15-LOX-1 is represented by LA, leading to the formation of 13(S)-HODE that activates PPAR-γ, an antimetastatic and anti-inflammatory factor in CRC [26,27].

The increase in expression of 15-LOX-1 found in the cell lines studied after GSE treatment confirms the antitumor effect exerted by these extracts, and the data obtained highlight a new mechanism of action through which GSEs inhibit colon tumorigenesis. Previously, we demonstrated the ability of GSEs to block cell migration and motility by inhibiting SCD1 and some components of the cytoskeleton [16]. The data obtained in this work show that the antiproliferative effect of GSEs also occurs through the induction of the expression of 15-LOX-1 that can be used for therapeutic purposes in CRC.

Cimen I. et al. have shown that 15-LOX-1 indirectly inhibits NF-kB through 13(S)-HODE-mediated PPAR-γ activation in HCT-116 and HT29 CRC cell lines, thus blocking cell proliferation [26]. Moreover, again in HCT-116 and HT29 cell lines, the expression of 15-LOX-1 reduced the ability of cells to adhere to fibronectin, thus inhibiting cell motility [42]. 

Previous in vitro studies have shown that grape extracts can act differently on proliferation and apoptotic pathways [43,44]. These different biological effects of GSEs could depend both on the type of cancer cell and on the different polyphenolic content of grape extracts [43,45,46]. In fact, it is known that there are cell lines more sensitive to treatment with polyphenols than others in relation to cellular differentiation degree. Moreover, certain flavonoids and non-flavonoids contained in grape extracts can act together synergistically to give particular antiproliferative effects on cancer cells [46,47]. These considerations suggest that the quality of the polyphenolic content in a grape cultivar is an important factor that must be considered.

### 2.4. Autumn Royal and Egnatia GSEs Induce Cell Morphological Changes

**Figure 4:**
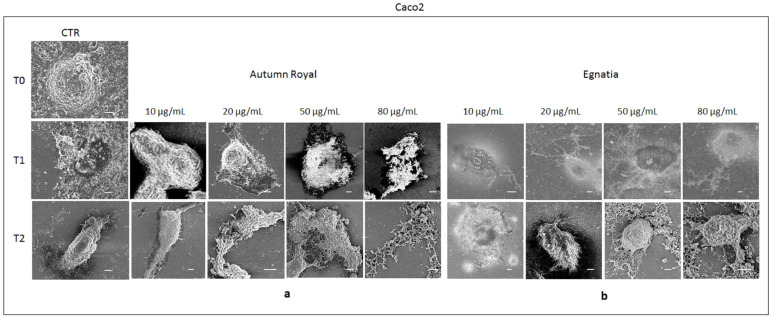
(**a**) Representative field emission scanning electron microscope (FE-SEM) micrographs (scale bar 5 µm, acquisition voltage 3 kV) of Caco2 cell line treated with increasing concentrations (10, 20, 50, 80 µg/mL) of Autumn Royal GSE after 24 (T1) and 48 (T2) h of incubation; (**b**) Representative FE-SEM micrographs (scale bar 5 µm, acquisition voltage 3 kV) of Caco2 cell line treated with increasing concentrations (10, 20, 50, 80 µg/mL) of Egnatia GSE after 24 (T1) and 48 (T2) h of incubation. The FE-SEM micrographs were selected as representative of a series of images collected on each sample. Untreated cells were used as control (CTR) at T0, T1, and T2. Each experiment was performed in triplicate.

**Figure 5:**
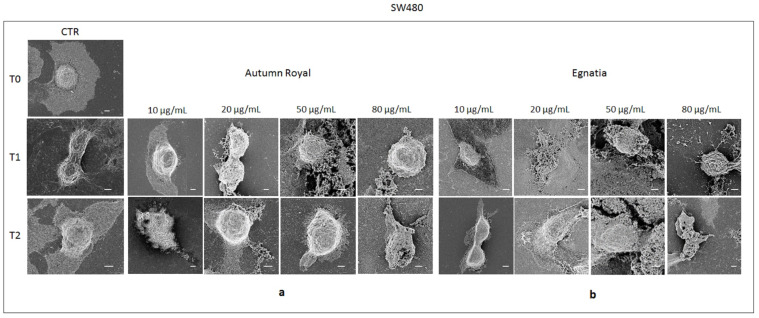
(**a**) Representative field emission scanning electron microscope (FE-SEM) micrographs (scale bar 5 µm, acquisition voltage 3 kV) of SW480 cell line treated with increasing concentrations (10, 20, 50, 80 µg/mL) of Autumn Royal GSE after 24 (T1) and 48 (T2) h of incubation; (**b**) Representative FE-SEM micrographs (scale bar 5 µm, acquisition voltage 3 kV) of SW480 cell line treated with increasing concentrations (10, 20, 50, 80 µg/mL) of Egnatia GSE after 24 (T1) and 48 (T2) h of incubation. The FE-SEM micrographs were selected as representative of a series of images collected on each sample. Untreated cells were used as control (CTR) at T0, T1, and T2. Each experiment was performed in triplicate.

Figure 4 and Figure 5 show the cell morphology of Caco2 and SW480 cell lines, respectively, treated with increasing concentrations of Autumn Royal and Egnatia GSEs (10, 20, 50, and 80 µg/mL) for 24 (T1) and 48 h (T2), analyzed by field emission scanning electron microscope (FE-SEM). To highlight the cellular morphological differences before and after each time GSEs exposure, untreated cells were used as control (CTR) at T0, T1, and T2 (Figure 4 and Figure 5).

Caco2 CTR cells appeared firmly adherent and covered with abundant microvilli, with a visible cytoplasm and at the center a notable nucleus region without shrinkage (Figure 4). Both at T1 and T2, treatment with Autumn Royal GSE induced visible characteristic morphological changes, such as shrinkage of membrane cells, starting from low concentrations of extract (10 µg/mL), showing a typical state of cell suffering (Figure 4a). In comparison to CTR, the increase of the Autumn Royal concentration induced evident and characteristic changes in the cells, as the cytoplasmic contraction and cell membrane collapse. Moreover, at the highest concentrations of Autumn Royal GSE, it was no longer possible to distinguish cell structures (Figure 4a). As regards the treatment with Egnatia GSE, at T1, the maximum concentration (80 µg/mL) was necessary to observe clear signs of apoptosis, while at T2, already at 50 µg/mL, membrane blebbing and cell shrinkage were noted (Figure 4b).

The FE-SEM micrographs reported in Figure 5 show the untreated controls (CTR) of the SW480 cell line that appeared flat and adherent to the substrate with an evident central nucleus. The morphological changes induced by Autumn Royal GSE on SW480 cells were visible at T1 starting from the highest treatment concentrations (50 and 80 µg/mL), given that at the lowest concentrations the cells were morphologically similar to the untreated cells (Figure 5a). At T2, the proapoptotic effect of the polyphenols contained in Autumn Royal GSE was appreciated, starting from the lowest concentrations (10 μg/mL) (Figure 5a). At the concentration of 80 μg/mL of GSE, the cellular structures did not appear very detailed and appreciable, when compared to the CTR; in addition, the apparent break of the surface of the cell membrane caused cell death (Figure 5a). Both at T1 and T2, SW480 cells treated with low concentrations of Egnatia GSE were visibly adherent with a round and abundant cytoplasm (Figure 5b). Furthermore, these cells were connected with neighboring cells and extended in all directions. The higher concentrations of GSE (50 and 80 μg/mL) induced characteristic changes, as well as the reduction of cell cytoplasm and a decrease of surface microvilli, which led to cellular apoptosis (Figure 5b).

To our knowledge, this is the first study describing cell morphological changes induced by polyphenols using FE-SEM micrographs. A previous study of Wang S. et al. demonstrated the ability of Trollius chinensis flavonoids to induce apoptosis in human breast cancer MCF-7 cells using SEM analysis [48]. In these cells treated with high concentrations of flavonoids, the microvilli on the cellular surface completely disappeared and cell membranes collapsed. Other morphological changes, in particular cell shrinkage and membrane blebbing, have been found in HCT-15 colon cancer cells after treatment with diet-derived gallic acid [49]. However, our study confirms previous data obtained about antiproliferative and proapoptotic effects in human colon cancer cell lines treated with both GSEs [28] and, for the first time, demonstrates that the beneficial effects of GSE polyphenols are also due to their ability to induce morphological changes in cancer cells, preventing their growth and proliferation.

## 3. Materials and Methods 

### 3.1. Preparation of the GSEs

Table grapes cultivar Autumn Royal, a seedless black grape variety, and Egnatia, a new red seedless genotype, were planted and grown in Apulia region at the Research Center for Viticulture and Enology of the Council for Agricultural Research and Economy (CREA-VE, Turi, BA, Italy). Grape samples were harvested at maturity in summer 2019 and berries were randomly collected and frozen at −20 °C. Approximately 100 frozen berries were manually peeled. To prepare the extracts, 250 mg of dry skin powder were mixed with 5 mL extraction solution of ethanol:water:hydrogen chloride 37% (70:30:1 *v*/*v*/*v*). After 24 h of complete darkness, the mixture was centrifuged, and the supernatant recovered, concentrated in a SpeedVac concentrator (Savant® SPD131DDA, Thermo Fisher Scientific, Waltham, MA, USA) for 90 min at 25 °C and 1.5 atmospheres of pressure and analyzed.

### 3.2. Total Polyphenolic Content

Total phenolic content was determined by Folin–Ciocalteu micro scale protocol with slight modification, as previously described [50]. Briefly, 1 mL of water, 0.02 mL of extract sample, 0.2 mL of the Folin reagent, and 0.8 mL of 10% sodium carbonate solution were mixed and brought to 3 mL. The absorbance was measured at 765 nm after 90 min. Results were expressed as mg GAE/g dw using calibration curves with standard gallic acid.

### 3.3. UHPLC/QTOF Mass Spectrometry

The extracts were three-fold diluted with H_2_O/CH_3_CN 95:5 (*v*/*v*) and analyzed using an Ultra-High Performance Liquid Chromatography (UHPLC) Agilent 1290 Infinity coupled to Agilent 1290 Infinity Autosampler (G4226A) and Agilent 6540 accurate-mass Quadrupole-Time of Flight (Q-TOF) Mass Spectrometer (nominal resolution 40.000) with Dual Agilent Jet Stream Ionization source (Agilent Technologies, Santa Clara, CA, USA). For each sample, two analyses in both positive and negative ionization mode by recording data in full scan acquisition mode were performed. Chromatographic separation was performed by using a Zorbax reverse-phase column (RRHD SB-C18 3 × 150 mm, 1.8 µm) (Agilent Technologies) and mobile phase composed by (A) 0.1% *v*/*v* aqueous formic acid and (B) 0.1% *v*/*v* formic acid in acetonitrile. Gradient elution program: 5% B isocratic for 8 min, from 5% to 45% B in 10 min, from 45% to 65% B in 5 min, from 65% to 90% in 4 min, 90% B isocratic for 10 min. Flow rate: 0.4 mL/min; sample injection: 10 µL; column temperature: 35 °C. After each sample a blank composed by the two mobile phases 1:1 *v*/*v* was analyzed to check the absence of false positives.

QTOF conditions used: sheath gas nitrogen 10 L/min at 400 °C; dehydration gas 8 L/min at 350 °C; nebulizer pressure 60 psi; nozzle voltage 0 kV (negative mode) and 1 kV (positive mode); capillary voltage ± 3.5 kV in positive and negative ion modes. Signals in the *m*/*z* 100–1700 range at acquisition rate 2 spectra/s were recorded. Mass calibrations were performed with standard mix G1969-85000 (Supelco Inc., Bellefonte, WA, USA), residual error for the expected masses between ± 0.2 ppm. Negative ionization lock masses: TFA anion at *m*/*z* 112.9856 and HP-0921 at *m*/*z* 966.0007 (ion [M + HCOO]^−^); positive ionization lock masses: purine at *m*/*z* 121.0509 and HP-0921 at *m*/*z* 922.0098. MS/MS fragmentation of the parent ions selected in the *m*/*z* 100–1700 range by using collision energies between 20 and 60 eV. Acquisition rate: 2 spectra/s.

Data acquisition software Agilent MassHunter version B.04.00 (B4033.2). Data analysis performed by using Agilent MassHunter Qualitative Analysis software B.05.00 (5.0.519.0). The overall identification score of compounds was calculated by the weighted average of the isotopic pattern signals (exact masses, relative abundances, *m/z* distances: W_mass_ = 100, W_abundance_ = 60, W_spacing_ = 50). Targeted identification of metabolites was performed by using the homemade database *GrapeMetabolomics* [51].

### 3.4. Cell Culture and Treatment

Human colon adenocarcinoma derived Caco2 cell line (ATCC: HTB 37) (well-differentiated) (G1–2) (from adenocarcinoma) and SW480 cell line (ATCC: CCL 228) (poorly-differentiated) (G3–4) (from adenocarcinoma grades III–IV) were purchased from the American Type Culture Collection (ATCC) Cell Bank (Manassas, VA, USA). Cells were cultured in Roswell Park Memorial Institute (RPMI) 1640 medium, for Caco2 cells, and Dulbecco’s Modified Eagle Medium (DMEM) for SW480 cells. All cell culture medium and reagents were purchased from Gibco, Life Technologies Limited, Paisley, UK. Culture medium was supplemented with 10% fetal bovine serum (FBS), 2 mM glutamine, 100 U/mL penicillin, and 100 µg/mL streptomycin and incubated at 37 °C in a humidified atmosphere containing 5% CO_2_ in air. Autumn Royal and Egnatia GSEs were added to the medium at increasing concentrations dissolved in 100 µL of solvent, composed by ethanol:water:hydrogen chloride 37% (70:30:1 *v*/*v*/*v*) (Sigma Aldrich, Milan, Italy), whereas the cells of control group received the same amount of the solvent. The cells were then incubated at 37 °C in a humidified 5% CO_2_ incubator for 24 and 48 h.

### 3.5. Lipids Extraction and PUFAs Analysis

Cell membrane fatty acids were extracted after 48 h of Autumn Royal or Egnatia GSEs treatment at 20, 50, and 80 µg/mL of concentrations. Untreated cells were used as control. Lipids from cell lysate were extracted using the Folch extraction method with some modifications [52]. PUFA analysis was assessed by a gas chromatograph (ThermoFisher Scientific, Focus GC, Milan, Italy) using ChromQuest 4.1 software (Thermo Fisher Scientific, Focus GC, Milan, Italy), as previously described [16].

### 3.6. RNA Extraction and Quantitative Real-Time PCR

After 48 h of treatment with Autumn Royal or Egnatia GSEs (20, 50, and 80 μg/mL), total RNA was extracted from Caco2 and SW480 cells using the Qiagen RNeasy Mini Kit (Qiagen, Hilden, Germany), according to the manufacturer’s instructions. The control sample was represented by untreated cells. Samples were retro-transcribed and analyzed using real-time Polymerase Chain Reaction (PCR) for the evaluation of 15-LOX-1 and PPAR-γ expressions on a CFX96 Touch Real-Time PCR Detection System (Bio-Rad Laboratories, Hercules, CA, USA) according to the manufacturer’s instructions. Table 3 shows gene-specific primer sets used (Bio-Rad Laboratories); β-actin gene was chosen as the reference gene. The ΔΔCt method was used for relative quantification by CFX Manager software 2.1 (Bio-Rad Laboratories).

### 3.7. FE-SEM Investigation

Caco2 and SW480 cells were seeded on 1 cm^2^ silicon chips (Ted Pella Inc., Redding, CA, USA) at a density of 10^4^ cells per well in eight-well chamber slides and, at a sub-confluent density, untreated control cells (CTR) were fixed at T0. To observe cell morphological changes, cell lines were exposed to increasing concentrations of Autumn Royal and Egnatia GSEs (10, 20, 50, and 80 µg/mL) for 24 (T1) and 48 h (T2). Cell lines were treated as previously described [53]. Briefly, each experimental time had a CTR group to highlight the cellular morphological differences before and after each time GSE exposure. As regards fixation procedures, the cells were treated with 3% glutaraldehyde in PBS for 1 h at 4 °C and then incubated for 1 h at room temperature in 1% osmium tetroxide (OsO_4_). Samples were washed several times with 0.005 M of sodium cacodylate pH 7.2, and the dehydration was completed by passing the samples in increasing concentrations of acetone, from 20% to 100%, for 10 min each step. After completing the drying, samples were coated with a thin Au film using a sputter coater (208HR High Resolution Sputter Coater, Ted Pella Inc.). The fixed cells were observed by using a Zeiss Sigma FE-SEM (Carl Zeiss, Oberkochen, Germany), equipped with an in-lens secondary electron detector. The samples deposited on silicon chips were fixed to stainless-steel sample holders by using double-sided carbon tape. A uniform Au metal coating of few nanometers was deposited on the samples placed on silicon chips by using a turbomolecular pumped SC7620 Mini Sputter/Glow Discharge System of Quorum Technologies (Quorum Technologies Ltd, Lewes, UK). The overall FE-SEM measurements on the samples were acquired at constant Extra-High Tension (EHT) value of 3 kV and at working distance (WD) ranging from 1.8 to 3 mm. The FE-SEM micrographs presented for each experiment were selected as representative of a series of images collected on each sample. Each experiment was performed in triplicate.

### 3.8. Statistical Analysis

Data on total polyphenolic content of GSEs were analyzed by paired Student *t*-test. The significance of the differences between the control and each treated experimental group was evaluated with one-way analysis of variance (ANOVA) and Dunnett’s posttest. Differences were considered as statistically significant with a *p*-value < 0.05. Data, expressed as mean ± Standard Deviation (SD), were analyzed using STATA statistical software, version 15.1 (StataCorp, 4905 Lakeway Drive, College Station, TX 77845, USA).

## 4. Conclusions

Our in vitro data wanted to emphasize the effects of table grape polyphenolic compounds on some molecular mechanisms involved in tumorigenesis. Moreover, the lipidomic approach followed in this study provided valuable information for understanding the protective effect of two GSEs studied on human cell metabolism. Given their ability to influence cell morphology, the flavonoid and non-flavonoid compounds present in table grapes could become a promising dietary source for cancer prevention and treatment.

## Figures and Tables

**Table 1:** Catechol derivatives identified in Autumn Royal and Egnatia grape skin extracts (GSEs).

	*o*-Diphenol Compounds
Phenolic acids	shikimic acid, methyl gallic acid, 5-hydroxy-ferulic acid, gallic acid glucoside, *trans*-caffeoyltartaric acid
Flavonols and Flavanonols	quercetin glycosides, myricetin glycosides, laricitrin glycosides, taxifolin-pentoside, dihydroquercetin-3-*O*-rhamnoside
Flavan-3-ols and Proanthocyanidins	(+)-catechin/(−)-epicatechin, (epi)gallocatechins, (−)-epicatechin gallate, procyanidins/prodelphinidins
Anthocyanins	cyanidin glycosides, delphinidin glycosides, petunidin glycosides

**Table 2:** (**a**) Mean percentage of n-6 and n-3 Polyunsaturated Fatty Acids (PUFAs) in the Caco2 membrane cell line treated with increasing concentrations of Autumn Royal and Egnatia GSEs (20, 50, and 80 µg/mL) after 48 h of treatment; (**b**) mean percentage of n-6 and n-3 Polyunsaturated Fatty Acids (PUFAs) in the SW480 membrane cell line treated with increasing concentrations of Autumn Royal and Egnatia GSEs (20, 50, and 80 µg/mL) after 48 h of treatment. All data are expressed as the mean ± Standard Deviation (SD) of three consecutive experiments. *p*-value was determined by ANOVA with Dunnett’s post-test. * *p* < 0.05 versus the untreated control group (CTR).

**a**	**Caco2 PUFAs (%)**		**Autumn Royal**			**Egnatia**		
		**CTR**	**20 µg/mL**	**50 µg/mL**	**80 µg/mL**	**20 µg/mL**	**50 µg/mL**	**80 µg/mL**
*n-6 PUFAs*	linoleic acid (LA)	2.20 ± 0.16	2.89 ± 0.37 *	3.20 ± 0.33 *	4.60 ± 0.39 *	3.58 ± 0.18 *	4.14 ± 0.32 *	6.40 ± 0.39 *
	γ-linolenic acid (GLA)	0.14 ± 0.04	0.22 ± 0.07	0.12 ± 0.05	0.21 ± 0.03	0.18 ± 0.11	0.13 ± 0.04	0.15 ± 0.05
	dihomo-γ-linoleic acid (DGLA)	0.38 ± 0.10	0.44 ± 0.04	0.30 ± 0.05	0.29 ± 0.10	0.37 ± 0.08	0.34 ± 0.10	0.30 ± 0.14
	arachidonic acid (AA)	3.87 ± 0.51	4.27 ± 0.26	4.26 ± 0.42	4.25 ± 0.33	4.60 ± 0.64	3.52 ± 0.63	3.65 ± 0.61
*n-3 PUFAs*	α-linolenic acid (ALA)	0.19 ± 0.04	0.57 ± 0.09 *	0.81 ± 0.07 *	1.34 ± 0.21 *	0.64 ± 0.06 *	0.93 ± 0.07 *	1.74 ± 0.03 *
	eicosapentaenoic acid (EPA)	0.93 ± 0.06	1.03 ± 0.14	0.78 ± 0.08	0.89 ± 0.11	0.92 ± 0.13	0.90 ± 0.08	0.94 ± 0.11
	docosaepentaenoic acid (DPA)	1.32 ± 0.10	1.52 ± 0.06	1.26 ± 0.10	1.29 ± 0.09	1.58 ± 0.12	1.53 ± 0.13	1.52 ± 0.09
	docosaehenanoic acid (DHA)	3.21 ± 0.50	3.64 ± 0.37	3.06 ± 0.21	3.26 ± 0.41	3.54 ± 0.95	3.20 ± 0.67	3.63 ± 0.77
	Total PUFAs	12.89 ± 0.24	15.74 ± 0.51 *	14.24 ± 0.71 *	16.34 ± 0.44 *	15.73 ± 0.50 *	15.29 ± 0.70 *	18.88 ± 0.42 *
**b**	**SW480 PUFAs (%)**		**Autumn Royal**			**Egnatia**		
		**CTR**	**20 µg/mL**	**50 µg/mL**	**80 µg/mL**	**20 µg/mL**	**50 µg/mL**	**80 µg/mL**
*n-6 PUFAs*	linoleic acid (LA)	3.00 ± 0.36	3.91 ± 0.37 *	4.20 ± 0.41 *	4.36 ± 0.35 *	3.82 ± 0.26	4.69 ± 0.51 *	5.64 ± 0.63 *
	γ-linolenic acid (GLA)	0.20 ± 0.02	0.18 ± 0.01	0.21 ± 0.03	0.23 ± 0.02	0.16 ± 0.03	0.21 ± 0.03	0.22 ± 0.06
	dihomo-γ-linoleic acid (DGLA)	0.06 ± 0.02	0.04 ± 0.02	0.04 ± 0.02	0.06 ± 0.01	0.03 ± 0.01	0.11 ± 0.04	0.06 ± 0.06
	arachidonic acid (AA)	6.28 ± 0.36	5.92 ± 0.45	4.68 ± 0.32 *	4.41 ± 0.26 *	5.69 ± 0.37	4.73 ± 0.70 *	4.37 ± 0.38 *
*n-3 PUFAs*	α-linolenic acid (ALA)	0.15 ± 0.06	0.12 ± 0.07	0.15 ± 0.02	0.52 ± 0.06 *	0.32 ± 0.05 *	0.46 ± 0.09 *	0.75 ± 0.10 *
	eicosapentaenoic acid (EPA)	0.63 ± 0.14	0.51 ± 0.22	0.65 ± 0.20	0.73 ± 0.07	0.54 ± 0.06	0.77 ± 0.02	0.79 ± 0.02
	docosaepentaenoic acid (DPA)	2.21 ± 0.41	2.89 ± 0.11	2.53 ± 0.11	2.55 ± 0.11	2.82 ± 0.17	2.64 ± 0.35	2.57 ± 0.60
	docosaehenanoic acid (DHA)	4.60 ± 0.47	5.31 ± 1.03	5.04 ± 0.09	4.92 ± 0.78	5.05 ± 0.80	4.89 ± 0.53	4.60 ± 0.39
	Total PUFAs	18.58 ± 0.62	19.87 ± 0.14 *	18.00 ± 0.15	18.52 ± 0.49	19.51 ± 0.59	18.49 ± 0.49	19.30 ± 0.29

**Table 3:** Primers for quantitative real-time PCR.

Target Genes	Gene Symbol	Gene Aliases	ID Assay
*Arachidonate 15-lipoxygenase*	ALOX15	15-LOX-1, 15LOX-1	qHsaCED0045954
*Peroxisome proliferator-activated receptor gamma*	PPARG	CIMT1, GLM1, NR1C3, PPARG1, PPARG2, PPARgamma	qHsaCID0011718
*Actin, beta*	ACTB	PS1TP5BP1	qHsaCED0036269

## Supplementary Materials

The following are available online at https://www.mdpi.com/1420-3049/25/15/3352/s1, Figure S1A: Positive extract ion chromatogram (EIC) of M^+^ signals of the anthocyanins identified, Figure S1B: Negative extract ion chromatogram (EIC) of [M − H]^−^ signals of the phenolic compounds identified, Table S1: Main MS/MS fragments and mean signal intensity of the metabolites identified in the samples.
